# Traumatic Brain Injury Induces Nociceptin/Orphanin FQ and Nociceptin Opioid Peptide Receptor Expression within 24 Hours

**DOI:** 10.3390/ijms25031658

**Published:** 2024-01-29

**Authors:** Omar N. Al Yacoub, Yong Zhang, Panini S. Patankar, Kelly M. Standifer

**Affiliations:** Department of Pharmaceutical Sciences, University of Oklahoma College of Pharmacy, University of Oklahoma Health Sciences Center, Oklahoma City, OK 73117, USA; alyac004@umn.edu (O.N.A.Y.); yong-zhang@ouhsc.edu (Y.Z.); paninishrikant-patankar@ouhsc.edu (P.S.P.)

**Keywords:** traumatic brain injury, nociceptin/orphanin FQ (N/OFQ), nociceptin opioid peptide (NOP) receptor, cofilin-1, mitogen-activated protein kinases, controlled cortical impact, injury severity, knockout rat

## Abstract

Traumatic brain injury (TBI) is a major cause of mortality and disability around the world, for which no treatment has been found. Nociceptin/Orphanin FQ (N/OFQ) and the nociceptin opioid peptide (NOP) receptor are rapidly increased in response to fluid percussion, stab injury, and controlled cortical impact (CCI) TBI. TBI-induced upregulation of N/OFQ contributes to cerebrovascular impairment, increased excitotoxicity, and neurobehavioral deficits. Our objective was to identify changes in N/OFQ and NOP receptor peptide, protein, and mRNA relative to the expression of injury markers and extracellular regulated kinase (ERK) 24 h following mild (mTBI) and moderate TBI (ModTBI) in wildtype (WT) and NOP receptor-knockout (KO) rats. N/OFQ was quantified by radioimmunoassay, mRNA expression was assessed using real-time PCR and protein levels were determined by immunoblot analysis. This study revealed increased N/OFQ mRNA and peptide levels in the CSF and ipsilateral tissue of WT, but not KO, rats 24 h post-TBI; NOP receptor mRNA increased after ModTBI. Cofilin-1 activation increased in the brain tissue of WT but not KO rats, ERK activation increased in all rats following ModTBI; no changes in injury marker levels were noted in brain tissue at this time. In conclusion, this study elucidates transcriptional and translational changes in the N/OFQ-NOP receptor system relative to TBI-induced neurological deficits and initiation of signaling cascades that support the investigation of the NOP receptor as a therapeutic target for TBI.

## 1. Introduction

Traumatic brain injury (TBI) is a neurological condition caused by external forces that disrupt brain function and neurobehavioral outcomes of patients [1]. The primary injury from the trauma, which can be penetrating or non-penetrating, is usually classified as focal and may spread through secondary mechanisms to become a diffuse injury; this transition occurs in the acute phase following TBI [2]. A sequence of molecular, cellular, and physiological events is initiated following the primary injury including axonal damage, impact depolarization, excitatory neurotransmitter release, neuronal excitotoxicity, blood–brain barrier disruption, and local and diffuse inflammatory responses [3,4,5]. This sequence of events contributes to the development of impaired cerebral blood flow, vasogenic and cytotoxic edema, and increased intracranial pressure [3,4,5]. The diagnosis and management of TBI within the first 24 h following injury is essential for faster recovery [6,7]. TBI symptoms and recovery depend on injury severity (mild, moderate, or severe) using the Glasgow Coma Scale, magnetic resonance imaging, and severity and number of patient symptoms [4,5,8]. Management provided for TBI patients is palliative in nature and focuses on treating the clinical consequences of TBI [9]. The U.S. Food and Drug Administration (FDA) has not yet approved drugs for the treatment of TBI, which highlights the importance of identifying new therapeutic targets.

Glial fibrillary acidic protein (GFAP) and ubiquitin C-terminal hydrolase (UCH-L1) were approved in 2018 by the FDA as TBI serum biomarkers as a means to determine the necessity of ordering imaging tests for mild TBI (mTBI) cases [10]. The axonal damage protein, neurofilament light chain (NF-L), is another TBI biomarker established in the literature [11,12,13,14,15]. Upregulation and activation of a cytoskeleton-associated protein, cofilin-1, contributes to actin filament depolymerization, inflammation, and the oxidative stress response following TBI [16,17].

Several experimental animal models have been employed to study the molecular and physiological mechanisms of brain injury and to understand the pathological processes that are triggered by brain trauma [18,19]. Controlled cortical impact (CCI) injury is one of the most studied models in the TBI field [18,19]. It can produce different severities of TBI using a pneumatic or electromagnetic impact device with adjustable injury parameters to propel a rigid impactor onto the surgically exposed brain cortex at defined coordinates [20]. CCI produces structural and pathophysiological responses similar in nature to characteristics of clinical focal TBI [3,20].

The nociceptin opioid peptide (NOP) receptor, part of the opioid receptor superfamily [21,22,23,24,25,26,27], and its endogenous neuropeptide, N/OFQ, are widely expressed in the human body, including neurons, astrocytes, and microglia [28,29]. Acute increases in N/OFQ levels were found in cerebrospinal fluid (CSF) shortly after fluid percussion injury (FPI) [30,31,32] and CCI [33]. N/OFQ vasodilates cortical pial arteries under normal conditions but produces vasoconstriction following FPI and CCI [33,34,35]. The vasoconstrictive action of N/OFQ was reversed by pretreatment with an NOP receptor antagonist [33] or partial agonist [35]. Increases in brain tissue N/OFQ levels are found acutely following stab wound injury [36] and blast TBI (bTBI) [37] and sub-acutely post-CCI TBI in rats [38]. The FPI, CCI, and bTBI studies implicated mitogen-activated protein kinases (MAPKs) in the cerebral vasoconstrictive effects of N/OFQ following TBI [33,37,39,40]. Though N/OFQ levels increased in ipsilateral brain tissue, 8 days post-CCI, they were unchanged in serum and CSF at that time [38]. We also demonstrated that NOP receptor-knockout (KO) rats recovered from CCI-induced neurobehavioral changes more quickly and completely over the subsequent 8 days [38]. The goal of this study was to examine neurological and biochemical changes that arise within the first 24 h after mild and moderate CCI TBI to determine how quickly N/OFQ and NOP receptor levels change in brain tissue and modify signaling pathways involved in TBI. Findings from this study along with our previous findings will add the important 24 h transition point that reflects two levels of injury severity to the temporal profile of changes in N/OFQ, NOP receptors, injury markers, and signaling proteins following CCI TBI.

## 2. Results

### 2.1. Neurological Scores on Day 1 Confirm Injury Severity; Moderate TBI Prolongs Righting Reflex Time in WT and KO Rats

**Figure 1:**
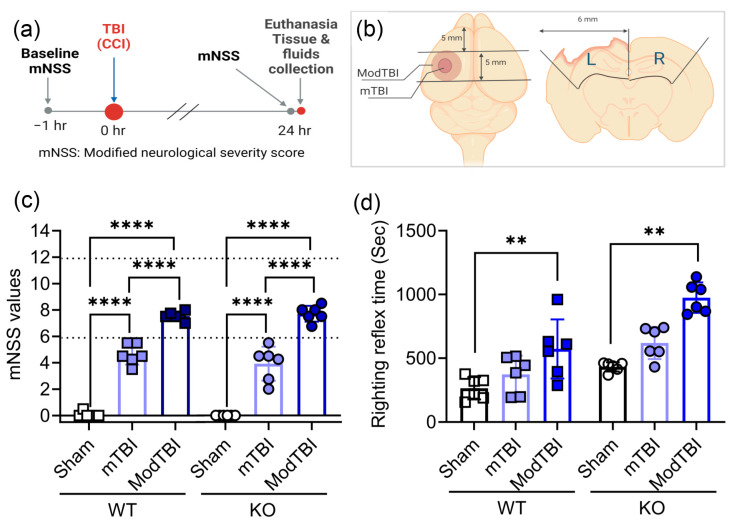
Neurological deficits 24 h post-TBI were injury severity- but not genotype-dependent; righting reflex time increased with ModTBI in both genotypes. Experimental design and site and size of injury are illustrated in (**a**,**b**), respectively. Red dots represent an invasive procedure done on animals. Grey dots represent the time point of non-invasive procedure (mNSS testing). The blue arrow color is meant to highlight the surgery itself. Slashed lines (//) inserted on the timeline show that the line here is shorter than it should be based on the 1 h length on the line relative to a full 24 h. Modified NSS values (**c**) and RR time (**d**) following sham or TBI surgery are shown as scatter plots with mean ± SD (*n* = 6 per group). The upper scores of mTBI and ModTBI injury severity are designated by dotted lines in (**c**) at 6 and 12, respectively. Data were analyzed by 2-way ANOVA with Tukey’s multiple comparisons test and significant differences are represented as ** *p* < 0.01, and **** *p* < 0.0001. ANOVA revealed a significant effect of injury severity on mNSS: (F (2, 30) = 375.0, *p* = < 0.0001) and on RR time (F (2, 30) = 18.06, *p* = < 0.0001). No effect of genotype or interaction between genotype and injury severity was noted for mNSS or RR. Panels (**a**,**b**) were created in BioRender.com (accessed on 26 January 2024).

Figure 1a,b illustrate the experimental design, size, and placement of the two levels of impact on the brain. The modified Neurological Severity Score (mNSS) was assessed for each rat prior to TBI (WT: sham 0 ± 0, mTBI 0.1 ± 0.2, ModTBI 0 ± 0; KO: sham 0.2 ± 0.3, mTBI 0.1 ± 0.2, ModTBI 0 ± 0) and 24 h post-TBI; no rats were excluded after the baseline test since all scored less than 1. All rats received a craniotomy and either no impact (sham) or mild (mTBI) or moderate (ModTBI) impact using the impact parameters described in the methods. The neurological scores of all rats were consistent with the mNSS scale: rats scored between 1 and 6 or 7 and 12 following TBI, consistent with mild or moderate injury (Figure 1c). No differences were noted between genotypes, as one expects for a primary injury, but scores significantly increased following mTBI (*p* < 0.0001) and ModTBI (*p* < 0.0001). Results were confirmed by two-way ANOVA performed on day 1 indicating a significant effect of injury severity, with no interaction between injury severity and genotype. Righting reflex (RR) time was measured after TBI surgery and was significantly prolonged in both WT and KO rats that received a ModTBI compared to sham (*p* < 0.01) or mTBI; RR time in mTBI rats did not differ from sham or between WT and KO genotypes (Figure 1d).

### 2.2. N/OFQ Levels Increase on the TBI-Injured Side of the Brain and in CSF of WT Rats 24 h Post-TBI

**Figure 2:**
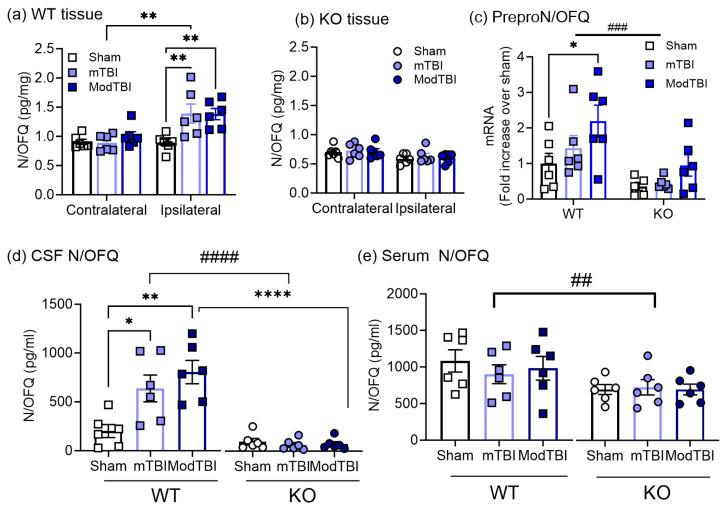
N/OFQ peptide and mRNA levels in tissue from WT and KO rat brains, and peptide in CSF and serum collected 24 h post-TBI. Data were analyzed using two-way ANOVA with Tukey’s post hoc test, and values are presented as mean ± SEM (n = 6 per group). N/OFQ peptide levels were determined in tissue from each side of the brain from WT (**a**) and KO males (**b**) with an RIA as described in Methods. Analysis revealed a significant interaction between brain hemisphere (ipsilateral or contralateral brain tissue) and injury severity (F (2, 30) = 4.365, *p* = 0.0217). A significant effect of genotype was found when % changes in ipsilateral tissue N/OFQ from sham were compared in WT and KO (F (1, 20) = 60.25, *p* < 0.0001). mRNA levels were determined by real-time PCR analysis from the same tissue and significant effects of genotype (**c**): (F (1, 29) = 15.41, *p* = 0.0005) and of TBI severity (F (2, 29) = 4.669, *p* = 0.0175) were noted. Levels of N/OFQ peptide were also quantified in CSF (**d**) and serum (**e**). Two-way ANOVA of CSF N/OFQ levels revealed an interaction between genotype and injury severity (F (2, 30) = 8.097, *p* = 0.0015). There was a significant effect of genotype on serum N/OFQ levels (F (1, 30) = 8.308, *p* = 0.0072). Significant differences from sham or contralateral side are represented by * *p* < 0.05, ** *p* < 0.01, and **** *p* < 0.0001; effect of genotype is denoted as ## *p* < 0.01, ### *p* < 0.001, and #### *p* < 0.0001 for serum, mRNA, and CSF, respectively.

Brain tissue from the injured (ipsilateral) and uninjured (contralateral) sides of each rat were subjected to radioimmunoassay to determine how and where N/OFQ levels changed 24 h after TBI in WT and NOP receptor-KO rats. N/OFQ was elevated in ipsilateral tissue following mild and ModTBI, compared to sham in WT male rats (Figure 2a), but not in tissue from the contralateral side. N/OFQ levels were unchanged by TBI in NOP receptor-KO tissue compared to sham (Figure 2b). A direct comparison of the percent change from sham in N/OFQ levels in the ipsilateral hemisphere of WT and KO brain revealed that N/OFQ levels increased in WT tissue following mTBI (55.9 ± 18.3) and ModTBI (55.0 ± 10.7)% and decreased in tissue from NOP receptor-KO rats following mTBI and ModTBI (−29.3 ± 5.6) and (−32.6 ± 3.9)%, respectively. This confirmed that TBI increased levels of N/OFQ at the site of injury significantly more in WT rats compared to their KO counterparts with the same injury severity (*p* < 0.0001). ModTBI increased preproN/OFQ mRNA compared to sham (* *p* < 0.05, Tukey’s multiple comparisons test), and mRNA increases in WT rats were significantly higher than in KO rats (*p* < 0.001), but no differences were noted between any of the injury groups in KO rats (Figure 2c).

N/OFQ levels were also measured in CSF and serum collected 24 h post-TBI. N/OFQ levels in CSF increased following mTBI (*p* < 0.05) and ModTBI (*p* < 0.01) compared to sham in WT rats, with a significant effect on injury severity (*p* = 0.0015). However, no differences were found between KO rat groups (Figure 2d). Levels of CSF N/OFQ in WT rat groups were higher than in KO groups (*p* = 0.0001). N/OFQ levels also were higher in WT rats than in KO rats with the same injury (Figure 2d). Serum N/OFQ levels did not differ between WT and KO rat groups (Figure 2e), as serum N/OFQ levels were higher in WT rats in general compared to KO rats (## *p* < 0.01; Figure 2e). These results confirm that N/OFQ levels are upregulated at the site of injury after TBI, and the upregulation is mediated through the NOP receptor.

### 2.3. NOP Receptor Protein and mRNA Expression Changes in Injured Brain Tissue of WT Rats 24 h Post-TBI

**Figure 3:**
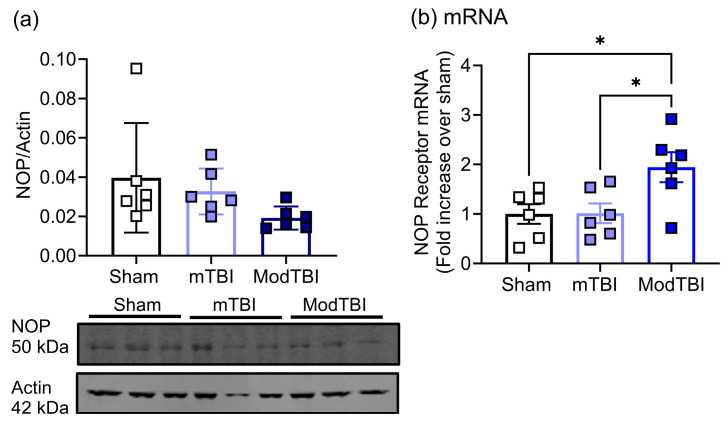
NOP receptor protein expression was unchanged (**a**) but mRNA expression increased (**b**) 24 h after ModTBI compared to sham and mTBI groups. (**a**) NOP expression was determined as described in Methods. Representative blots are shown below the graph. Kruskal–Wallis test indicates a significant effect of injury (**a**; *p* = 0.0249), but no differences between groups were found. Values are presented as mean ± SD (*n* = 6 per group; * *p* < 0.05). (**b**) NOP receptor gene expression from all groups was normalized to GAPDH and fold change in mRNA compared to sham was determined as described in Methods. Values are presented as mean ± SEM and compared using one-way ANOVA with Tukey’s multiple comparisons test.

The effect of TBI on NOP receptor protein expression was examined by immunoblotting, comparing tissue lysates from the ipsilateral hemispheres from WT sham-, mTBI-, and ModTBI-treated rats. Because the data were not normally distributed in all three groups, a Kruskal–Wallis test was used to determine if there was an effect of injury severity—and there was (* *p* = 0.0249). Although NOP receptor expression in tissue from ModTBI rats appeared lower than in sham rats, it did not quite reach significance with Dunn’s multiple comparisons test (*p* = 0.0602) 24 h post-TBI (Figure 3a).

However, ModTBI increased NOP receptor mRNA in ipsilateral tissue compared to that from sham and mTBI (Figure 3b, * *p* < 0.05).

### 2.4. Cofilin-1 and Extracellular Regulated Kinase (ERK) Activation Increased following TBI Compared to Sham

**Figure 4:**
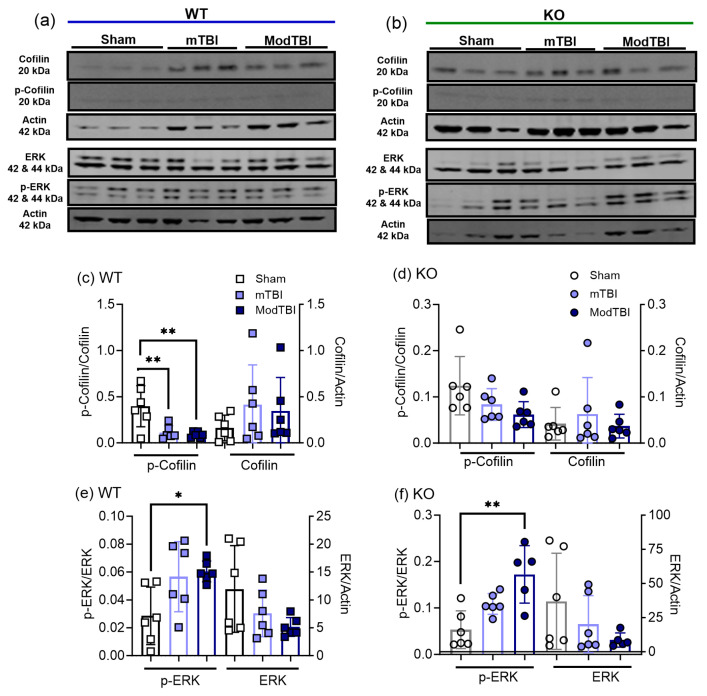
Changes in activation of cofilin-1 and ERK MAPK following TBI in tissue from WT and KO rat brains collected 24 hr post-TBI. Immunoblots are representative of p-cofilin-1, cofilin-1, p-ERK, and ERK immunolabeling from WT (**a**) and KO (**b**) brain tissue of 6 rats in each group 24 h post-TBI. Expression of p-cofilin and cofilin (WT (**c**) and KO (**d**)) and p-ERK and ERK (WT (**e**) and KO (**f**)) were quantified by densitometric analysis and normalized as described in Methods. One-way ANOVA was performed to assess differences and indicated significant group effects for WT p-cofilin/cofilin (F (2, 15) = 9.436; *p* = 0.002), WT pERK/ERK (F (2,15) = 4.864; *p* < 0.0235), and KO pERK/ERK (F (2, 14) = 11.05; *p* = 0.0013). Significant differences between groups were determined by Tukey’s multiple comparisons post hoc tests and are denoted by * *p* < 0.05, and ** *p* < 0.01. Values are presented as mean ± SD (*n* = 6 per group).

Cofilin-1 becomes activated when it is dephosphorylated [41,42]. Ratios of phospho-cofilin-1/cofilin-1 and cofilin-1/actin expression were calculated for immunoblots of tissue on the side of the brain receiving sham, mild, and ModTBI (ipsilateral). Phospho-cofilin-1 expression was significantly reduced in mTBI and ModTBI (*p* < 0.01) tissue from WT rats compared to those receiving sham TBI (Figure 4c), but no significant changes were noted in tissue from KO rat brains (Figure 4d). No significant differences in the expression of cofilin-1 between TBI and sham groups were found in WT (Figure 4c) or KO (Figure 4d) rats.

Phospho-ERK expression increased following moderate, but not mild, TBI in tissue from the ipsilateral hemisphere of WT (Figure 4e; *p* < 0.05) and KO (Figure 4f; *p* < 0.001) rats compared to sham. However, total ERK levels in tissues from WT and KO rats remained unchanged by trauma. This suggests that the activation of cofilin (denoted as a decrease in the phospho-cofilin/cofilin ratio) is more sensitive to brain trauma since it is stimulated following mTBI, while ERK requires a more severe injury (ModTBI) for activation.

### 2.5. Injury Markers NF-L, GFAP, and UCH-L1 Remained Unchanged in Ipsilateral Brain Tissue 24 h post-TBI

**Figure 5:**
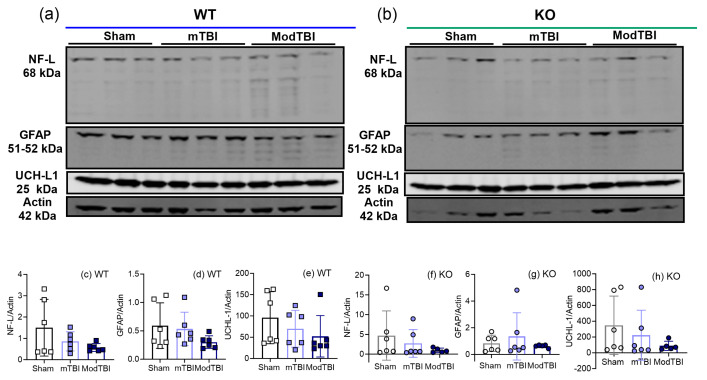
Protein expression of injury markers NF-L, GFAP, and UCH-L1 in ipsilateral tissue is not altered by TBI in WT or KO rats at 24 h post-TBI. Representative blots from WT and KO rat tissue are shown in (**a**) and (**b**), respectively. NF-L (**c**,**f**), GFAP (**d**,**g**), and UCH-L1 (**e**,**h**) expression were quantified by densitometric analysis of immunolabeled bands, and values normalized to actin loading control from the same lane. Data were analyzed by one-way ANOVA with Tukey’s post hoc test and values are presented as mean ± SD (*n* = 6 per group).

The effect of TBI on injury marker expression after 24 h in tissue from ipsilateral hemispheres from WT and KO rats also was examined by immunoblotting. No differences in NF-L (Figure 5c,f), GFAP (Figure 5d,g), and UCH-L1 (Figure 5e,h) expression between sham and TBI rats of either genotype were found.

### 2.6. Pearson Correlation Analysis Supports the Relationship between Changes in N/OFQ/NOP and Other Outcomes following TBI

**Figure 6:**
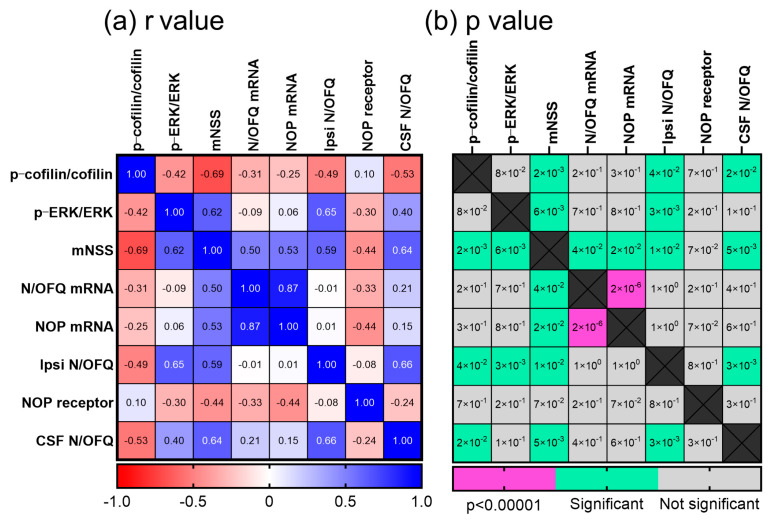
Pearson Correlation Analysis matrices of (**a**) r and (**b**) *p* values associated with outcomes that changed 24 h post-TBI in WT rats. Color coding of r values ranges from dark red (strong negative correlation) to light red, white or light blue (weak or no correlation) to dark blue (strong positive correlation. *p* values associated with those r values are coded to represent significance at *p* < 0.000001 (dark pink) or *p* < 0.05 to *p* < 0.00001 (green) and not significant (gray); *n* = 18 rats.

The highest correlation was found between NOP receptor mRNA and N/OFQ mRNA (Figure 6a,b). This positive correlation is consistent with increasing N/OFQ mRNA driving an upregulation of N/OFQ and NOP receptor mRNA, confirming the strong relationship between N/OFQ and NOP receptor expression. N/OFQ peptide and mRNA and NOP receptor mRNA strongly and positively correlated with mNSS values 24 h post-TBI, but NOP receptor protein levels correlated weakly with all other outcomes assessed. Tissue N/OFQ expression strongly correlated with both ERK and cofilin activation, though because cofilin activation is reflected as a decrease in p-cofilin, this appears as a negative correlation. Together, this suggests that increased N/OFQ levels resulting from TBI contribute to increased mNSS scores (consistent with increased neurological deficits) and increased cofilin and ERK activation.

## 3. Discussion

This study generated several important and novel findings to advance our understanding of N/OFQ-NOP receptor system dysregulation at 24 h following mTBI and ModTBI CCI. First, N/OFQ levels in CSF and ipsilateral tissue increased following mild and ModTBI in WT but not in NOP receptor-KO rats, and strongly correlated with increased neurological deficits. Second, mild and ModTBI increased activation of cofilin-1 in WT but not KO rats. Third, activation of ERK MAPK was evident in both WT and KO rat tissue only after moderate CCI impact.

General neurological function was determined by the mNSS test before and after TBI [43]. The severity of the intended CCI parameters aligned with the mNSS values obtained and confirmed reproducible mild and moderate primary injury that did not differ between genotypes (Figure 1c). These findings are consistent with previous studies following CCI, stab, and blast TBI that suggested that the NOP receptor does not contribute to the primary acute physical injury but that it modulates the severity and duration of the secondary injuries arising later [37,38], including activation of signaling pathways [36,37,44].

**Figure 7:**
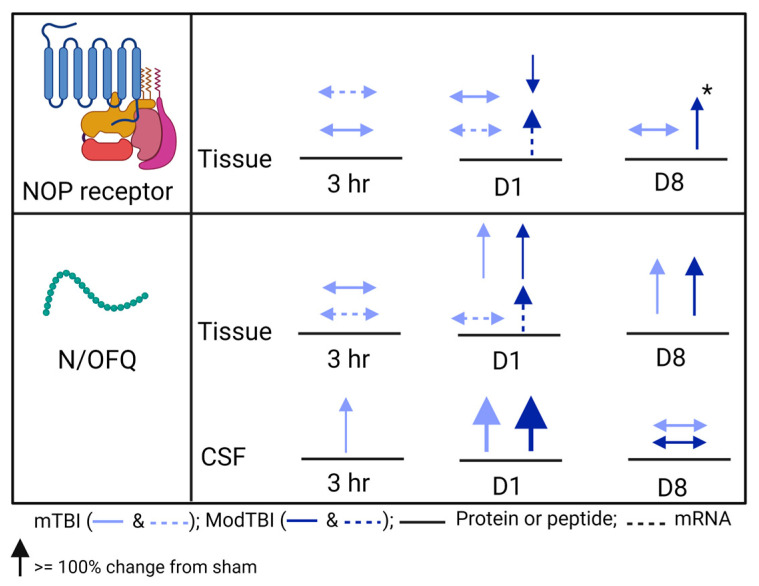
Schematic representation of CCI TBI-induced changes in N/OFQ and NOP receptor protein and mRNA as a function of injury severity and time. The color of the arrows matches the color of the data points on graphs for mild (light blue) and moderate (dark blue) TBI; ↑ arrows represent increase, ↓ arrows represent decrease, and ↔ represents no significant change compared to sham. The width of the arrow represents the size of the change in NOP or N/OFQ levels. Dashed arrows represent mRNA, while solid arrows represent protein or peptide levels. * represents change from contralateral (undamaged) tissue. Data noted in the 3 h [33] and 8-day [38] time points are from recent published publications; the day 1 data are from the current study on WT rats. This figure was created in BioRender.com (accessed on 22 January 2024).

Though several previous TBI studies reported upregulation of the N/OFQ-NOP receptor system following TBI, none of them comprehensively examined protein, peptide, and mRNA level changes as a function of TBI severity [33,35,36,37,38]. In this study, we report increases in N/OFQ levels in the injured brain tissue and CSF following mTBI and ModTBI on day 1 post-TBI in WT rats (Figure 2a,d). Together with results from 8 days [38] and 3 h post-TBI [33], this current study at 24 h post-TBI helps us to draw the following conclusions regarding CCI TBI-induced temporal changes in N/OFQ levels in WT male rats that are schematically represented in Figure 7: First, N/OFQ levels in CSF increased within 3 h and stayed elevated at least for 24 h before returning to levels found in sham rats on day 8 post-TBI. Second, N/OFQ levels in pericontusional tissue were upregulated at 24 h post-TBI to the same extent in both mild and ModTBI, and this was accompanied by increased mRNA with Mod TBI. They remained elevated through day 8 post-TBI, but by day 8, they were higher after ModTBI than mTBI [38], perhaps because of the upregulated mRNA on day 1. Third, N/OFQ levels in serum did not change between 3 h and 8 days post-TBI. Fourth, none of these changes were found in the tissue or biological fluids of NOP receptor-KO rats post-TBI. Fifth, NOP receptor mRNA levels increased after ModTBI at 24 h, which ultimately resulted in increased NOP receptor protein levels by 8 days following ModTBI. Lastly, the correlation of mNSS values with N/OFQ levels in CSF and tissue in WT, but not KO, rats indicates an association between increased N/OFQ levels and the severity of neurological deficits.

The dynamic changes in NOP receptor and N/OFQ mRNA 24 h post-TBI likely represent an acute response to the trauma and explain the elevated N/OFQ in injured tissue only. Therefore, we can conclude from our previous finding on day 8 [38] that the upregulation of NOP receptor protein expression occurs between day 1 and day 8 following ModTBI in male rats. These findings are important to improve our understanding of temporal changes in the N/OFQ-NOP receptor system following TBI and the contribution that functional NOP receptors make in N/OFQ upregulation following TBI.

We previously reported increased cofilin-1 activation 3 h post-mTBI in ipsilateral tissue [33]. In this study, changes in expression of cofilin-1 and p-cofilin-1 were assessed in tissue collected from male rats of both genotypes 24 h post-TBI. Both mild and ModTBI induced the activation of cofilin-1 by decreasing its phosphorylation 24 h post-TBI in WT rats (Figure 4c). Interestingly, mild and ModTBI did not increase cofilin-1 activation in NOP receptor-KO rats (Figure 4d). Tissue N/OFQ levels correlated with p-cofilin/cofilin-1 values in ipsilateral tissue on day 1, which were absent 3 h post-mTBI [33]. This is consistent with the increased vasoconstrictive action of N/OFQ in tissue post-TBI that produces an ischemic response [16,17], resulting in cofilin-1 activation.

Increased phosphorylation (e.g., activation) of ERK MAPK was detected in the ipsilateral brain tissue of both WT (Figure 4e) and NOP receptor-KO rats (Figure 4f) following ModTBI compared to sham. Several studies reported that ERK was activated after TBI, including by glutamate [37,39]. N/OFQ activated ERK following other types of TBI [37,39,40], including 3 h after CCI, so it is not surprising to see ERK activated at 24 h post-TBI in WT and in NOP receptor-KO rats. These findings agree with the idea that the N/OFQ-NOP receptor system is not the only contributor to ERK MAPK activation at this early time point post-TBI. However, ERK activation also increases ppN/OFQ mRNA [44,45,46], so it likely plays a dual role of vasoconstriction and N/OFQ upregulation, which is supported by the strong positive correlation between p-ERK expression and N/OFQ levels in WT, but not KO, tissue. These results, together with our previous findings at 3 h post-TBI [33], indicate that the ERK activation in pericontusional brain tissue following mild TBI CCI begins around 3 h post-TBI and lasts less than 24 h, while ModTBI-induced ERK activation is maintained for at least 24 h.

NOP receptor transcription is much less well understood [45]. However, NOP receptor transcripts are increased during neurodevelopment by N/OFQ [46], and NOP receptor upregulation following bTBI was reduced by acute treatment with an NOP receptor antagonist [37], consistent with our findings in this paper.

Early and sub-acute elevations (2 h to 5 days) of UCH-L1 [47,48] and NF-L [49,50] levels were reported in serum and CSF post-TBI, with GFAP elevated as early as 3 days post-TBI [51,52], but we found no changes in tissue 3 h post-TBI [33] or at the 24 h time point in this study (Figure 5). However, a large, severity-dependent increase in the expression of GFAP and NF-L in ipsilateral tissue was found 8 days following CCI TBI in male and female rats [38]. This suggests that changes in the injury markers GFAP and NF-L in tissue appear after 24 h and before 8 days post-TBI.

In conclusion, our findings provide novel information about acute transcriptional and translational changes in N/OFQ and the NOP receptor following two different severities of TBI. Changes in N/OFQ-NOP expression and dysregulation following CCI TBI should be considered along with findings from previous reports using different models such as bTBI [37], stab wound injury [36], and fluid percussion injury [35] in designing preclinical and clinical studies to test potential NOP receptor ligands as therapeutics for TBI. The time course suggests that the treatment window with these modulators would span at least 24 h after injury and provides a window within which to test efficacy.

## 4. Materials and Methods

### 4.1. Animals

Homozygous Oprl1-TGEM^®^ KO (ORL1−/− or NOP receptor-KO) [53,54] rats from a Wistar Han background were originally obtained from Transposagen (Lexington, KY, USA), and a colony was maintained in the University of Oklahoma College of Pharmacy animal facility. The NOP receptor-KO genotype was confirmed by analysis of ear punch samples (Transnetyx, Cordova, TN, USA). WT Wistar Han rats were purchased from Charles River Labs (Wilmington, MA, USA) and were allowed to acclimate for 7 days after arrival. Male WT and KO rats (175–200 g, 9–14 weeks) were housed in the animal facility under a 12 h light: 12 h dark cycle (lights on at 0600) with free access to food and water. Experimental protocols were approved by the institutional animal care and use committee (IACUC), and studies were conducted in compliance with animal welfare act (AWA) regulations, Animal Research: Reporting of In Vivo Experiments (ARRIVE) guidelines 2.0 [55], and other federal statutes relating to animals and experiments involving animals. Rats were randomly assigned to receive either sham, mild, or moderate TBI surgery, and the experimenter was masked to the injury groups. The following data were collected from 6 injury groups (WT sham, KO sham, WT mTBI, KO mTBI, WT ModTBI, KO ModTBI), with *n* = 6 rats per group.

### 4.2. Induction of TBI

CCI was performed as previously described [38] based upon previous reports [56,57] with validation of mild and moderate TBI severity using neurological deficit assessments explained below. Anesthetized rats (4% isoflurane with medical air induction; 2.5 to 3% maintenance) underwent stereotaxic surgery with a midline incision, exposure of the skull using a retractor, and assignment of Bregma as a reference using the stereotaxic manipulator (Stoelting Co., Wood Dale, IL, USA). Control (sham) injury animals received a 7–9 mm craniotomy without impact while keeping the dura mater intact over the left parietal cortex.

TBI rats received a craniotomy followed by a mild or moderate controlled cortical impact with stereotaxic coordinates (1.8 mm posterior, 3.0 mm lateral to the left of the bregma) using the Impact One device (Leica Biosystems, Richmond, IL, USA) and the following actuator settings: Impactor flat tip diameter (2 mm in mTBI, 5 mm in ModTBI), velocity (3 m/s for mTBI, 5 m/sec for ModTBI), dwell time (100 m for mTBI, 200 ms for ModTBI), and impact depth (4 mm for mTBI, 3 mm for ModTBI). After each sham or CCI injury, the bone flap was sealed in place with sterile bone wax, and wounds were sutured with staples and tissue adhesive followed by topical antibiotic ointment treatment. Righting reflex (RR) time, the amount of time it took for the animal to stand on all four feet after isoflurane was discontinued, was recorded for each rat. Body temperature was maintained at 37 °C throughout the surgery.

### 4.3. mNSS

The mNSS [58] assesses general neurological function and was employed to validate baseline and the severity of injury 24 h post-surgery. Evaluation indices include a battery of individual assessments including: motor (raising rat by the tail (0–3); walking on floor (0–3)), sensory (proprioceptive test (0–1); visual and tactile test (0–1)), Reflex (pinna reflex (0–1); corneal reflex (0–1); startle reflex (0–1)), resting movement (seizures, myoclonus, myodystony (0–1)), and beam balance (0–6) tests; normal function receives a value of 0. Neurological deficit severity is categorized based on cumulative score: Severe = 13–18, moderate = 6–12, mild = 1–6 [58]. Rats lacking neurological deficits score less than 1.

### 4.4. Processing and Collection of Biofluid and Brain Tissue Samples

After cardiac exsanguination, blood was stored at room temperature for 30 min. The serum sample was supernatant collected after centrifugation at 5000× *g*, 4 °C for 5 min [59]; it was flash frozen in 250 µL aliquots. CSF (100~200 µL) was collected from the direct insertion of a 26-gauge needle into the cisterna magna. Brain tissue samples: Rat brains were extracted and dissected using a matrix brain slicer (Zivic Instruments) to include separate 5 mm sections of ipsilateral (left) and contralateral (right) tissue (cortex, corpus callosum, and hippocampus), as illustrated in Figure 1b. Brain tissue was then homogenized and allocated for radioimmunoassay (~200 µL), immunoblotting (80 µL), and qPCR (20 µL).

### 4.5. Radioimmunoassay (RIA)

The N/OFQ content of CSF, serum, and tissue samples was determined in duplicate according to the manufacturer’s protocol using an RIA kit obtained from Phoenix Pharmaceuticals (Belmont, CA, USA). Peptide extraction from the brain tissue samples was performed as described in the manufacturer’s protocol. The concentration of total soluble proteins in the brain tissue extract was determined by the bicinchoninic acid method (Pierce TM BCA protein assay kit, ThermoFisher Scientific Inc., Waltham, MA USA). Total N/OFQ immunoreactivity (IR) was calculated and expressed as pg/mL in CSF and serum samples and as pg/mg for tissue samples.

### 4.6. Immunoblotting

Frozen tissue homogenates were thawed and treated with cell lysis buffer (50 mM Tris PH 7.5, 0.5 M NaCl, 50 mM NaF, 10 mM EDTA, 2 mM EGTA, 1% Triton X-100, 2 mM Na3VO4, 10 µM Na4P2O7, 250 µM PMSF) with a freshly added protease and phosphatase inhibitor cocktail (Santa Cruz Biotechnology, Inc., Dallas, TX, USA) for 30 min on ice prior to centrifugation at 14,000× *g* at 4 °C for 20 min. The protein concentration of the supernatants was determined as described above, prior to solubilization in 4X sample loading buffer (LI-COR Biosciences, Lincoln, NE, USA) heated to 65 °C for 20 min. Soluble protein samples (20 µg of total protein) were resolved by Novex™ WedgeWell™ 8–16% Tris-Glycine gradient gels (Thermo Fisher Scientific Inc., Waltham, MA, USA), transferred to nitrocellulose membranes, and probed with antibodies directed against the following proteins: NOP receptor (bs-0181R, 1:500; Bioss Inc., Woburn, MA, USA), GFAP (GPCA-GFAP, 1:4000; EnCor Biotechnology, Gainesville, FL, USA), UCH-L1 (sc-271639, 1:200), NF-L (sc-20012, 1:200), and cofilin (sc-376476, 1:200) from Santa Cruz Biotechnology, Inc., Dallas, TX, USA; ERK1/2 (4696S, 1:2000) and phospho-ERK1/2 (4370S, 1:500 and phospho-cofilin (Ser3) (#3313S; 1:250) from Cell Signaling Technology, Inc. (Danvers, MA, USA); and actin (A3853, 1:2000; Sigma-Aldrich, Inc, St. Louis, MO, USA). Blots were incubated in primary antibody overnight at 4 °C, rinsed 4 times, and probed with one of the following secondary antibodies for 1 h at room temperature: IRDye^®^ 800CW goat anti-rabbit (1:10,000), IRDye^®^ 680CW donkey anti-rabbit (1:10,000), IRDye^®^ 680CW donkey anti-mouse (1:10,000), IRDye^®^ 800CW donkey anti-mouse (1:10,000), IRDye^®^ 800CW donkey anti-goat (1:10,000), or IRDye^®^ 680CW goat anti-mouse (1:10,000), purchased from LI-COR Biosciences (Lincoln, NE, USA). Blots were rinsed, images were captured, and band density was analyzed using the Odyssey^®^ CLx Infrared Imaging System (LI-COR Biosciences, Lincoln, NE, USA). Band density was normalized to the loading control, actin, in the corresponding lane using Image Studio™ Lite image processing software v5.2 (LI-COR Biosciences, Lincoln, NE, USA). Quantification of the GFAP bands included all GFAP breakdown product bands.

### 4.7. Real-Time PCR

TriPure reagent (Sigma-Aldrich, MO, USA) was immediately added to brain tissue following dissection for mRNA extraction and stored at −80 °C. cDNA was synthesized using Super-Script III Reverse Transcriptase (Sigma-Aldrich, St. Louis, MO, USA). For real-time PCR analysis, duplicate cDNA samples were mixed with PowerUp™ SYBR™ Green Master Mix (Applied Biosystems, Foster City, CA, USA) and 125 nM forward and reverse primers of target genes (rat GAPDH Fwd: 5′-ACC CAG AAG ACT GTG GAT GG-3′, Rev: 5′-CAC ATT GGG GGT AGG AAC AC-3′; rat NOP Fwd: 5′-GTT CAA GGA CTG GGT GTT CAG CCA GGT AGT-3′; rat NOP Rev: 5′-TGC TGG CCG TGG TAC TGT CTC AGA ACT CTT-3′; rat preproN/OFQ Fwd: 5′-TGC ACC AGA ATG GTA ATG TG-3′, Rev: 5′-TAG CAA CAG GAT TGT GGT GA-3′ (Sigma-Aldrich) in a StepOnePlus Real-time PCR system (Applied Biosystems). The GAPDH gene was used as an internal standard to which the expression of other genes in each sample was normalized. Data were analyzed using the comparative Ct method. To determine fold change over sham, each 2^-DeltaCt value was divided by the average of all sham values [60], and groups were analyzed by one-way ANOVA (NOP receptor) or two-way ANOVA (preproN/OFQ) to determine the effects of genotype and/or injury severity.

### 4.8. Data Analysis

GraphPad Prism v. 10.1.2 software was used for data analysis and to prepare graphs (GraphPad Software, La Jolla, CA, USA). RR time, mNSS, and immunoblotting data are expressed as mean ± SD; N/OFQ and preproN/OFQ mRNA data are expressed as mean ± SEM. Statistical comparisons of mNSS, RR time, N/OFQ levels, and preproN/OFQ mRNA data were performed by two-way ANOVA when two variables affected the results: time and severity injury, brain side and injury severity, or genotype and injury severity. Tukey’s post hoc analyses were performed following ANOVA as recommended by the software. A one-way ANOVA was performed to analyze immunoblotting results for WT and KO samples separately since the tissue samples were not obtained at the same time. Results were considered significant if *p* < 0.05. All data were subjected to D’Agostino and Pearson or Shapiro–Wilk normality tests prior to analysis. Those groups that failed the normality test (*p* < 0.05) were subjected to an outlier test (ROUT; Q = 1%), as recommended by Prism software, v10.1.2 to determine whether the outlier was responsible for the failed normality test. If non-normally distributed data did not result from an outlier, they were analyzed by non-parametric analysis. Pearson’s Correlation Analysis (two-tailed) was performed with the following data aligned from each WT and KO rat: CSF N/OFQ, mNSS values, and l p-cofilin-1, p-ERK, N/OFQ and N/OFQ mRNA from ipsilateral brain tissue. NOP receptor and NOP receptor mRNA were included in the WT analysis. R values were computed for every pair of y data sets to generate a correlation matrix and heat maps of r and *p* values.

## Data Availability

The raw data supporting the conclusion of this article will be made available by the authors, without undue reservation.

## Abbreviations

Traumatic brain injury (TBI); Nociceptin/Orphanin FQ (N/OFQ); Nociceptin opioid peptide (NOP) receptor; Extracellular regulated kinase (ERK); Mitogen-activated protein kinases (MAPKs); Controlled cortical impact (CCI); Righting reflex (RR); Modified neurological severity score (mNSS); Mild TBI (mTBI); Moderate TBI (ModTBI); Wildtype (WT); Knockout (KO); Neurofilament light (NFL); Glial fibrillary acidic protein (GFAP); Radioimmunoassay (RIA); Ubiquitin C-terminal hydrolase L1 (UCH-L1).
